# Development and validation of a tool to assess the risk of QT drug-drug interactions in clinical practice

**DOI:** 10.1186/s12911-020-01181-3

**Published:** 2020-07-23

**Authors:** Florine A. Berger, Heleen van der Sijs, Matthijs L. Becker, Teun van Gelder, Patricia M. L. A. van den Bemt

**Affiliations:** 1 Department of Hospital Pharmacy, Erasmus MC, University Medical Center Rotterdam, Doctor Molewaterplein 40, 3015 GD Rotterdam, the Netherlands; 2 Pharmacy Foundation of Haarlem Hospitals, Haarlem, the Netherlands; 3 Department of Clinical Pharmacy, Spaarne Gasthuis, Haarlem, the Netherlands; 4 Department of Clinical Pharmacy & Toxicology, Leiden University Medical Center, Leiden, the Netherlands; 5 Department of Clinical Pharmacy and Pharmacology, University Medical Center Groningen, Groningen, the Netherlands

**Keywords:** Risk factors, ROC curve, Decision support systems, clinical, Drug interactions, Sensitivity and specificity

## Abstract

**Background:**

The exact risk of developing QTc-prolongation when using a combination of QTc-prolonging drugs is still unknown, making it difficult to interpret these QT drug-drug interactions (QT-DDIs). A tool to identify high-risk patients is needed to support healthcare providers in handling automatically generated alerts in clinical practice. The main aim of this study was to develop and validate a tool to assess the risk of QT-DDIs in clinical practice.

**Methods:**

A model was developed based on risk factors associated with QTc-prolongation determined in a prospective study on QT-DDIs in a university medical center inthe Netherlands. The main outcome measure was QTc-prolongation defined as a QTc interval > 450 ms for males and > 470 ms for females. Risk points were assigned to risk factors based on their odds ratios. Additional risk factors were added based on a literature review. The ability of the model to predict QTc-prolongation was validated in an independent dataset obtained from a general teaching hospital against QTc-prolongation as measured by an ECG as the gold standard. Sensitivities, specificities, false omission rates, accuracy and Youden’s index were calculated.

**Results:**

The model included age, gender, cardiac comorbidities, hypertension, diabetes mellitus, renal function, potassium levels, loop diuretics, and QTc-prolonging drugs as risk factors. Application of the model to the independent dataset resulted in an area under the ROC-curve of 0.54 (95% CI 0.51–0.56) when QTc-prolongation was defined as > 450/470 ms, and 0.59 (0.54–0.63) when QTc-prolongation was defined as > 500 ms. A cut-off value of 6 led to a sensitivity of 76.6 and 83.9% and a specificity of 28.5 and 27.5% respectively.

**Conclusions:**

A clinical decision support tool with fair performance characteristics was developed. Optimization of this tool may aid in assessing the risk associated with QT-DDIs.

**Trial registration:**

No trial registration, MEC-2015-368.

## Background

QTc-prolongation is known as a risk factor for developing ventricular arrhythmias such as Torsade de Pointes (TdP), which may eventually lead to sudden cardiac death. Therefore, a prolonged heart-rate corrected QT(c) interval is used as electrocardiogram (ECG) marker for an increased risk of TdP; and thus a prolonged QTc-interval should be avoided in patient care as a part of risk minimization [1–3].

QTc-prolongation is defined as a QTc-interval > 450 ms in males and > 470 ms in females according to the European Medicine Agency guidelines [4, 5]. However, arrhythmias are frequently associated with QTc-intervals exceeding 500 ms. [6–8] A prolonged QTc-interval often represents a delayed ventricular repolarization. Roden et al. introduced a theory where some physiological mechanisms create a buffer to maintain normal ventricular repolarization, the so-called repolarization reserve. Several risk factors and genetic predisposition can reduce this repolarization reserve causing abnormalities in the ventricular repolarization [9, 10]. Consequently, multiple risk factors are frequently present in case reports describing patients who developed serious QTc-prolongation or TdP [11, 12].

Several drugs are also responsible for developing QTc-prolongation known as drug-induced QTc-prolongation. Currently, over 190 drugs are associated with QTc-prolongation according to the CredibleMeds® QT drug lists of the Arizona Center for Education and Research on Therapeutics (AZCERT). AZCERT categorizes QTc-prolonging drugs into three categories representing the level of certainty on the risk of TdP. More than 50 drugs are categorized as drugs with a known risk of TdP [13]. Many of these drugs such as antibiotics and antidepressants are widely used in clinical practice. QTc-prolonging drugs are not further classified with respect to the extent of QTc-prolongation. Also, the exact risk of developing QTc-prolongation when using a combination of QTc-prolonging drugs is unknown. For healthcare professionals, such as physicians and pharmacists, it is difficult to decide whether or not it is safe to proceed treating a patient with combinations of two or more QTc-prolonging drugs, and in whom additional checks of ECGs after treatment initiation are needed.

Other risk factors include hypokalemia, hypomagnesemia, heart diseases (i.e. ischemic heart diseases, heart failure, and arrhythmia such as atrial fibrillation), and renal impairment. Also, demographic risk factors such as an older age, female sex and genetic predisposition are associated with QTc-prolongation [2, 12, 14–16]. However, the impact of these risk factors on the extent of QTc-prolongation is largely unknown, which makes it challenging to identify patients at risk for QTc-prolongation.

In the Netherlands, QT-DDI alerts are generated by the Computerized Physician Order Entry (CPOE) systems when two or more QTc-prolonging drugs with a known risk of TdP are combined. QT-DDI alerts are generated according to the so-called ‘G-Standard’, a Dutch drug database which supports the different processes in healthcare, such as prescription, dispensing, ordering, reimbursement, and decision support [17]. The current guidelines incorporated in the ‘G-Standard’ regarding QT-DDIs suggest to substitute or remove one of the interacting agents or perform routine ECG monitoring. As a result, first-line treatments are frequently not adhered to when one of the interacting agents is substituted, especially in primary care where ECG monitoring is often not feasible. In tertiary care, low adherence to guidelines result in many overridden DDI alerts by physicians [18]; and ECG monitoring is rarely performed when QT-DDI alerts are overridden [19, 20]. With the increasing number of QTc-prolonging drugs, QT-DDI alerts will reduce the physician responsiveness to this particular type of alert, also known as *alert fatigue*. The use of a smart algorithm which generates specific alerts will reduce alert fatigue in clinical practice.

## Methods

### The aim, design and setting

The aim of this study was to develop and validate a clinical decision support tool to assess the risk of QT-DDIs in clinical practice.

A prospective, observational study design was chosen to identify potential risk factors of QTc-prolongation in patients using two or more QTc-prolonging drugs with a known risk of TdP as part of their usual care. This study was performed in the Erasmus University Medical Center Rotterdam, the Netherlands. An external validation was performed on retrospective data obtained from the Spaarne Gasthuis, a general teaching hospital with locations in Haarlem and Hoofddorp, the Netherlands.

### Identification of potential risk factors and data collection

In the prospective study, patients (≥18 years) admitted to the Erasmus University Medical Center from September 2015 to March 2016 using two or more QTc-prolonging drugs with ‘a known risk of TdP’ [13] were included. A standard twelve-lead resting ECG (paper speed 25 mm s^− 1^, amplitude 10 mm mV^− 1^ and sampling rate 250 Hz) was recorded using the ***Mortara® ELI-350 ECG device (Milwaukee, Wisconsin, USA)*** at the estimated time of peak concentration (T_max_) of the lastly added drug, or at the longest T_max_ in case both drugs were started at the same time. Exclusion criteria included ECGs with a QTc-interval > 700 ms or <  300 ms, or with a ventricular rate (VR) > 180 beats per minute (bpm) or < 40 bpm as such ECGs do not allow reliable measurements of QTc-intervals; however, these ECGs were not present in our cohort. Patients with a congenital long QT syndrome, an implantable cardioverter-defibrillator (ICD) or a pacemaker were excluded. Also, patients with a left or right bundle branch block (LBBB/RBBB), atrial fibrillation or other ECG abnormalities due to cerebral pathology, ischemia or bigeminy were excluded as these comorbidities interfere with the QTc-interval.

The following data were prospectively collected from the electronic patient health record (Elpado, Rotterdam, the Netherlands): general patient characteristics including comorbidities and the medical condition at time of the ECG recording as well as the dose of the interacting drugs. The serum sodium (mmol L^− 1^), potassium (mmol L^− 1^), magnesium (mmol L^− 1^), and calcium (mmol L^− 1^) levels were collected within 5 days before or after the ECG recording, collecting the measurement closest to the ECG recording. Calcium levels were corrected for albumin levels [21]. The estimated glomerular filtration rate (eGFR, mL min^− 1^) using the Modification of Diet in Renal Disease (MDRD) formula, creatinine (μmol L^− 1^), aspartate transaminase (ASAT, U L^− 1^), alanine aminotransferase (ALAT, U L^− 1^), and bilirubin (μmol L^− 1^) were also obtained within 5 days before or after the ECG recording. Concomitant medication data were collected from the CPOE system Medicator (Computer Sciences Corporation (CSC) Healthcare Group, Leiden, the Netherlands) within 8 h prior to the ECG recording [22]. The QT-intervals were manually measured, preferably from lead II, from the onset of the QRS-complex to the end of the T-wave using the tangent method. The QT-interval was adjusted for heart rate using the Bazett (QTc = QT/√RR) and Fridericia (QTc = QT/^3^√RR) formula [23, 24].

### Statistical analysis

Data were analyzed using Statistical Package for Social Science (SPSS, IBM SPSS Statistics version 21.0, Armonk, NY, United States). The QTc-interval was dichotomized as either prolonged or not prolonged (QTc > 450 ms for males and QTc > 470 ms for females) [8]. Univariate logistic regression analysis was performed to determine potential risk factors, due to small sample size no multivariate logistic regression analysis was performed. Effect sizes were presented as odds ratios (OR) with their corresponding 95% confidence intervals (95% CI). A risk score of 1 to 3 points was assigned to potential risk factors based on their log odds ratios: ≤ 0.44 = 1 point; 0.45–0.94 = 2 points; ≥ 0.95 = 3 points.

### Literature review on risk factors

A small dataset will not identify all potential risk factors and a model can benefit from the information of previous studies. Therefore, a literature review was performed [25, 26]. Additional risk factors from this review were incorporated into the model when they are easily obtainable in tertiary and primary care. Large cohort studies were retrieved from the database Medline. Study population, cases of QTc-prolongation, formula to correct for heart rate, cut-off values of QTc-prolongation and the statistically significant risk factors associated with QTc-prolongation were evaluated. The level of evidence was determined based on the level of significance in the studies evaluated. Also, reviews on drug-induced QTc-prolongation were included to select relevant risk factors [2, 6, 12]. A risk score of 1 or 2 points was assigned to the additional risk factors based on the level of evidence.

### External validation

The validity of the model was assessed in an independent dataset from a general teaching hospital to evaluate model performance and clinical usefulness. All ECGs that were recorded in routine clinical practice of ambulatory and hospitalized patients using two or more QTc-prolonging drugs between January 21st, 2013 and October 10th, 2016 were extracted from the hospital information system EPIC (Madison, WI, USA) using SAP Crystal Reports (Walldorf, Germany). All ECGs were standard twelve-lead resting ECGs with automatically calculated heart rates (RR), QT-intervals and QRS-complexes by the MUSE Cardiology Information System. Firstly, for ECGs with QRS-complexes > 120 ms, the QT-intervals were corrected using the following equation: QT adjusted = QT – (QRS – 120). The QT-intervals were then corrected for heart rate using the Bazett and Fridericia formula [24]. A prolonged QTc-interval was defined as QTc > 450 ms for males and > 470 ms for females identical to the development cohort. Because arrhythmias are often associated with a QTc > 500 ms, we performed a post-hoc analysis in which QTc-prolongation was defined as QTc > 500 ms [6, 7]. ECGs with a QTc-interval > 700 ms or <  300 ms, or a VR > 180 bpm or < 40 bpm were excluded. Each patient was only included once using the first ECG available. Of these patients, data on risk factors included in the risk model were extracted such as age, sex, serum potassium (mmol L^− 1^), eGFR based on the MDRD (mL min^− 1^), cardiac comorbidities (based on Anatomical Therapeutic Chemical Classication (ATC) C01), hypertension (based on ATC C02, C03, C07 – C09), diabetes mellitus (based on ATC A10) concomitant medication such as loop diuretics (based on ATC C03CA) and the use of QTc-prolonging drugs at time of ECG recording [27].

The QTc-intervals > 450 ms in males and > 470 ms in females, and as post-hoc analysis QTc-intervals > 500 ms as measured by the ECG were taken as outcome measures, to which the performance of the model was compared. The model’s potential clinical usefulness was assessed by its’ ability to distinguish patients with and without QTc-prolongation. The discriminative ability was quantified with receiver operating characteristics (ROC)-analyses, also known as concordance statistic (C-statistic). Cut-off points for the model were selected by maximizing the difference between sensitivity and 1 minus specificity. The primary focus was maximizing the sensitivity to identify low-risk patients, while keeping specificity at an acceptable level. Therefore, a cut-off value with a sensitivity of > 75% in order to increase the specificity as much as possible is accepted. Specificity, sensitivity, accuracy, the false omission rate and the Youden’s index were calculated as these are the most relevant parameters for assessing clinical usability. Data are presented as mean with their standard deviation (SD) and median with their interquartile range (IQR).

## Results

### Study population

**Fig. 1:**
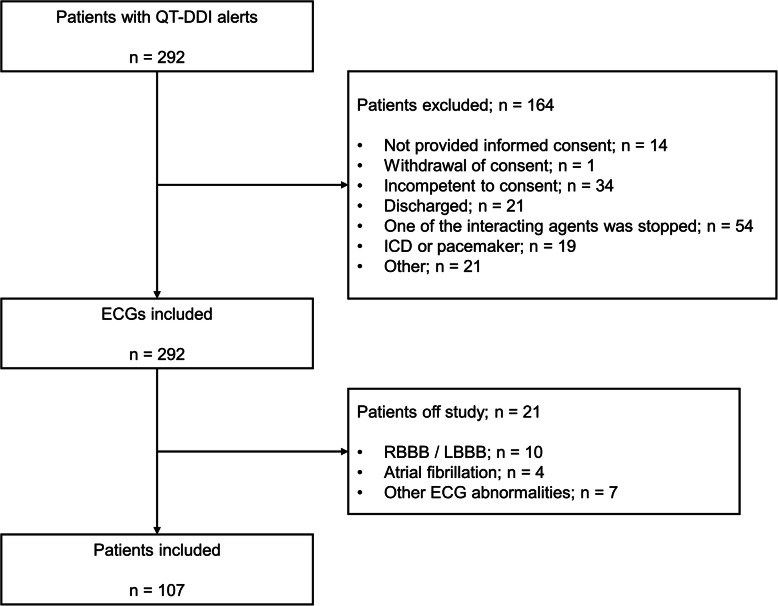
Flowchart of patient inclusion of the development cohort. Abbreviations: DDI, drug-drug interactions; RBBB/LBBB, right or left bundle branch block

In total, 107 patients were included in the development dataset, and 1579 patients were included in the validation dataset. The flowchart of inclusion is shown in Fig. 1 and the patients characteristics in Table 1. The median age of the validation cohort was significantly higher than the median age of the development cohort (77 to respectively 56 years old).

**Table 1:** Patient characteristics of the development and validation cohort

Patient characteristics	Development cohort (*n* = 107)	Validation cohort (*n* = 1579)	*P*-value
Age (years), median; IQR	56.0; 23.0	77.0; 17.0	< 0.001^a^
≤ 50, n (%)	38 (35.5)	94 (6.0)	< 0.001^b^
51–75, n (%)	60 (56.1)	646 (40.9)	
≥ 76, n (%)	9 (8.4)	839 (53.1)	
Female, n (%)	46 (43.0)	731 (46.3)	0.507^b^
Comorbidities, n (%)			
Cardiac comorbidities	17 (15.9)	664 (42.1)	< 0.001^b^
Hypertension	30 (28.0)	1064 (67.4)	< 0.001^b^
Diabetes Mellitus	13 (12.1)	357 (22.6)	0.011^b^
eGFR (MDRD) (≤ 50 ml min^−1^), n (%)	9 (8.4)	439 (27.8)	< 0.001^b^
Hypokalemia (< 3.5 mmol L^− 1^), n (%)	5 (4.7)	158 (10.0)	0.023^b^
> 2 QTc-prolonging drugs ^c^, n (%)	7 (6.5)	101 (6.4)	0.953^b^
Loop diuretics, n (%)	23 (21.5)	400 (25.3)	0.376^b^

*Abbreviations: eGFR, estimated Glomerular Filtration Rate; IQR, Interquartile Range*

^a^ Independent t test

^b^ Chi-square test

^c^ QTc-prolonging drugs with a known risk of TdP [13]

Missing values: Development cohort: eGFR, *n* = 2; K^+^, *n* = 1; Validation cohort: eGFR, *n* = 311; K^+^, *n* = 266; Validation cohort: eGFR, *n* = 310; K^+^, *n* = 265

### Identification of risk factors

Of the 107 patients (43% female, median (IQR) age 56 (23)( years) included, twenty-seven (25.2%) showed a prolonged QTc-interval on the ECG during treatment with two or more QTc-prolonging drugs. In none of these patients the QTc-interval was prolonged to more than 500 ms. The results of the univariate logistic regression analyses using the Bazett formula are presented in Table 2*.*

**Table 2:** The association of several risk factors with QTc-prolongation in the development cohort (Bazett formula)

Potential determinant	QTc- prolongation *n* = 27	No QTc-prolongation *n* = 80	OR	95% CI
Age (in years) median; IQR	58.0; 14.0	54.5; 23.0	1.02	0.99–1.05
≤ 25, n (%)	1 (3.7)	4 (5.0)	*Ref.*	*Ref.*
26–50, n (%)	5 (18.5)	28 (35.0)	0.71	0.07–7.79
51–75, n (%)	17 (63.0)	43 (53.8)	1.58	0.17–15.19
≥ 76, n (%)	4 (14.8)	5 (6.3)	3.20	0.25–41.21
Female gender, n (%)	4 (14.8)	42 (52.5)	0.16	0.05–0.50*
Caucasian race, n (%)	26 (96.3)	74 (92.5)	2.11	0.24–18.35
BMI (kg m^2–1^) ^a^, mean ± SD	25.7 ± 4.3	27.3 ± 5.3	0.93	0.85–1.03
Clinical departments, n (%)				
Medical Units	14 (51.9)	69 (86.3)	*Ref.*	*Ref.*
Surgical Units	9 (33.3)	8 (10.0)	5.55	1.82–16.86*
Cardiac Units	4 (14.8)	3 (3.8)	6.57	1.32–32.66*
Comorbidities				
Myocardial infarction	1 (3.7)	1 (1.3)	3.04	0.18–50.32
Heart failure	1 (3.7)	3 (3.8)	0.99	0.10–9.91
Arrhythmia	6 (22.2)	6 (7.5)	3.52	1.03–12.07*
Hypertension	12 (48.1)	17 (21.3)	3.44	1.36–8.68*
Diabetes Mellitus	5 (18.5)	8 (10.0)	2.05	0.61–6.89
COPD/Asthma	1 (3.7)	11 (13.8)	0.24	0.03–1.96
Hematological malignancies	12 (44.4)	55 (68.8)	0.36	0.15–0.89*
Hepatic dysfunction ^b^, n (%)				
Increased ASAT (> 170 / 150 U L^− 1^)	–	3 (3.8)	–	–
Increased ALAT (> 220 / 160 U L^− 1^)	–	1 (1.3)	–	–
Increased bilirubin (> 16 μmol L^− 1^)	2 (7.4)	16 (20.0)	0.33	0.07–1.55
eGFR ≤50 ml min^− 1^ (MDRD) ^c^, n (%)	3 (11.1)	6 (7.5)	1.50	0.35–6.47
Electrolyte disturbances ^d^, n (%)				
Hyponatremia (< 136 mmol L^− 1^)	2 (7.4)	19 (23.8)	0.25	0.06–1.17
Hypokalemia (< 3.5 mmol L^− 1^)	2 (7.4)	3 (3.8)	2.03	0.32–12.83
Hypocalcemia (< 2.2 mmol L^− 1^)	7 (25.9)	17 (21.3)	1.29	0.39–4.22
Hypomagnesemia (< 0.7 mmol L^− 1^)	4 (14.8)	10 (12.5)	1.33	0.34–5.29
Concomitant medication, median; IQR	8.0; 4.0	8.0; 4.0	1.02	0.86–1.20
Loop diuretics, n (%)	8 (29.6)	15 (18.8)	1.83	0.67–4.95
QTc-prolonging drugs ^e^, n (%)				
0	4 (14.8)	11 (13.8)	*Ref.*	*Ref.*
1	6 (22.2)	28 (35.0)	0.59	0.14–2.50
≥ 2	17 (63.0)	41 (51.3)	1.14	0.32–4.09

*Abbreviations: Ref. reference value; eGFR, estimated glomular filtration rate; IQR, interquartile range; BMI, body mass index; SD, standard deviation; OR, odds ratio; 95% CI, 95% confidence interval*

* Statistically significant

^a^ Missing values: BMI: no QTc, *n* = 1

^b^ Missing values: ASAT/ASAT: QTc, *n* = 5; no QTc, *n* = 7; Bili: QTc, *n* = 4; no QTc, *n* = 9

^c^ Missing values: eGFR: no QTc, *n* = 2

^d^ Missing values: Na^+^/K^+^: no QTc, *n* = 1; Ca^2+^: QTc, *n* = 12; no QTc, *n* = 38; Mg^2+^: QTc, *n* = 14; no QTc, *n* = 40

^e^ Other than the QTc-prolonging drugs with a known risk of TdP [13]

A history of arrhythmia (OR 3.52; 95% CI 1.03–12.07) and hypertension (OR 3.44; 95% CI 1.36–8.86) were significantly associated with QTc-prolongation. The use of loop diuretics (OR 3.65; 95% CI 1.18–11.25) was also identified as a potential risk factor for QTc-prolongation when using the Fridericia formula. Risk score points were assigned to the potential risk factors based on their odds ratios (Table 3).

**Table 3:** Risk scores assigned to potential risk factors based on their Log OR

	*Bazett formula*			*Fridericia formula*		
Predictors	Log OR	Score	OR (95% CI)	Log OR	Score	OR (95% CI)
Age (in years)						
≤ 25	*Ref.*	*Ref.*	*Ref.*	*Ref.*	*Ref.*	*Ref.*
26–50	− 0.15	0	0.71 (0.07–7.79)	–	–	–
51–75	0.20	1	1.58 (0.17–15.19)	–	–	–
≥ 76	0.51	2	3.20 (0.25–41.21)	–	–	–
Arrhythmia	0.55	2	3.52 (1.03–12.07)	–	–	–
Hypertension	0.54	2	3.44 (1.36–8.68)	0.77	2	5.92 (1.92–28.27)
Loop diuretics	–	–	–	0.56	2	3.65 (1.18–11.24)

*Abbreviations: Ref, reference value; OR, odds ratio; 95% CI, 95% confidence interval*

### Review from literature on additional risk factors

The literature review included reviews and cohort studies with 8453 patients in total [2, 15, 28–33]. Of the 8453 patients, 1772 patients (21%) showed QTc-prolongation assuming the studies were sufficiently powered to determine potential risk factors [15, 28–31]. In most studies [15, 29, 30], hypokalemia was highly associated with QTc-prolongation with a significance level of *p* < 0.001. Due to the level of significance and the number of studies, severe hypokalemia (≤ 2.5 mmol L^− 1^) was allocated 2 points and moderate hypokalemia (2.6–3.4 mmol L^− 1^) was allocated 1 point in the model. Female sex was associated with QTc-prolongation in three studies [15, 30, 31] with a significance level of *p* < 0.05, so 1 point was assigned to female sex [2, 32]. The comorbidities renal failure and diabetes mellitus showed limited evidence in the studies with significance levels of *p* < 0.05 [30, 32]. For QTc-prolonging drugs eliminated primarily by renal excretion, an impaired renal function can cause accumulation and toxicity of the QTc-prolonging drugs. Hemodialysis patients are also at increased risk for QTc-prolongation due to electrolyte abnormalities [34]. In addition, long-term glycemic variabilities in patients with diabetes mellitus, can induce QTc-prolongation; both comorbidities were therefore assigned 1 point in the model [35, 36]. The use of QTc-prolonging drugs with a known risk of TdP was highly associated with QTc-prolongation in several studies (*p* < 0.01) [2, 11, 15, 29, 30]. As the model focused on patients using two or more QTc-prolonging drugs with a known risk of TdP, QTc-prolonging drugs with a known risk of TdP were incorporated in the model with 1 point. The QTc-prolonging drugs with a possible and conditional risk of TdP were not found to be associated with QTc-prolongation in multiple cohort studies, and were therefore not taken into account. The final clinical risk model, is presented in Table 4.

**Table 4:** The risk model

Risk factors	Score
Age (in years)	
51–75	1
≥ 76	2
Female gender	1
Comorbidities	
Cardiac comorbidities	2
Hypertension	2
Diabetes Mellitus I and II	1
eGFR ≤50 mL min^− 1^ (MDRD)	1
Potassium levels	
≤ 2.5 mmol L^− 1^	2
2.6–3.4 mmol L^− 1^	1
Loop diuretics	2
QTc-prolonging drugs with a known risk of TdP ^a^	1

^a^ Classified according to the CredibleMeds® QT drug lists [13]

### External validation

In total, 6361 ECGs of patients using two or more QTc-prolonging drugs were extracted from the hospital information system EPIC (Madison, WI, USA). The ECGs included in the validation dataset belonged to 2514 unique patients. Because perioperative patients and patients admitted to the intensive care unit (ICU) were not included in the development cohort, we excluded QT-DDI alerts in the validation cohort with propofol as these alerts concerned perioperative and ICU patients. Also, 2 ECGs were excluded because the heart rates were > 180 bpm.

Eventually, the validation cohort consisted of 3891 ECGs of 1579 unique patients. The mean QTc-interval of the first ECG available was 453.7 ms. In total, 655 (41.5%) ECGs showed a prolonged QTc-interval defined as > 450/470 ms (m/f). The mean ± SD risk score of patients with a QTc-interval > 450/470 ms (m/f) was 7.4 ± 2.5; the mean ± SD risk score of patients with a normal QTc-interval was 7.2 ± 2.5.

**Fig. 2:**
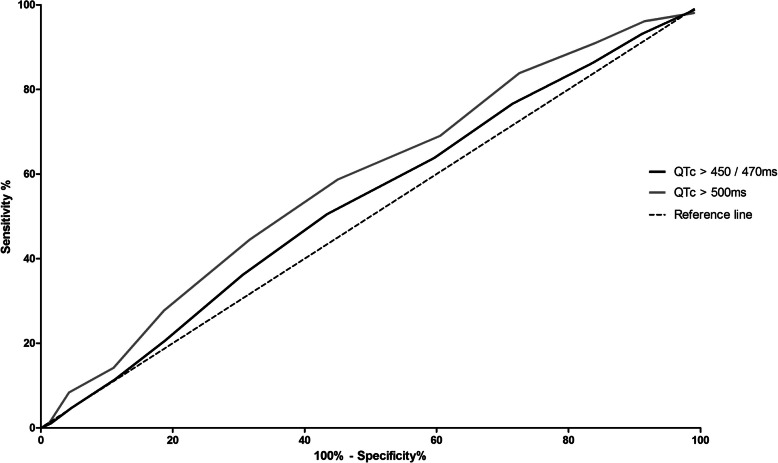
ROC-curves (> 450/470 ms and > 500 ms) of the risk model in the external validation

**Fig. 3:**
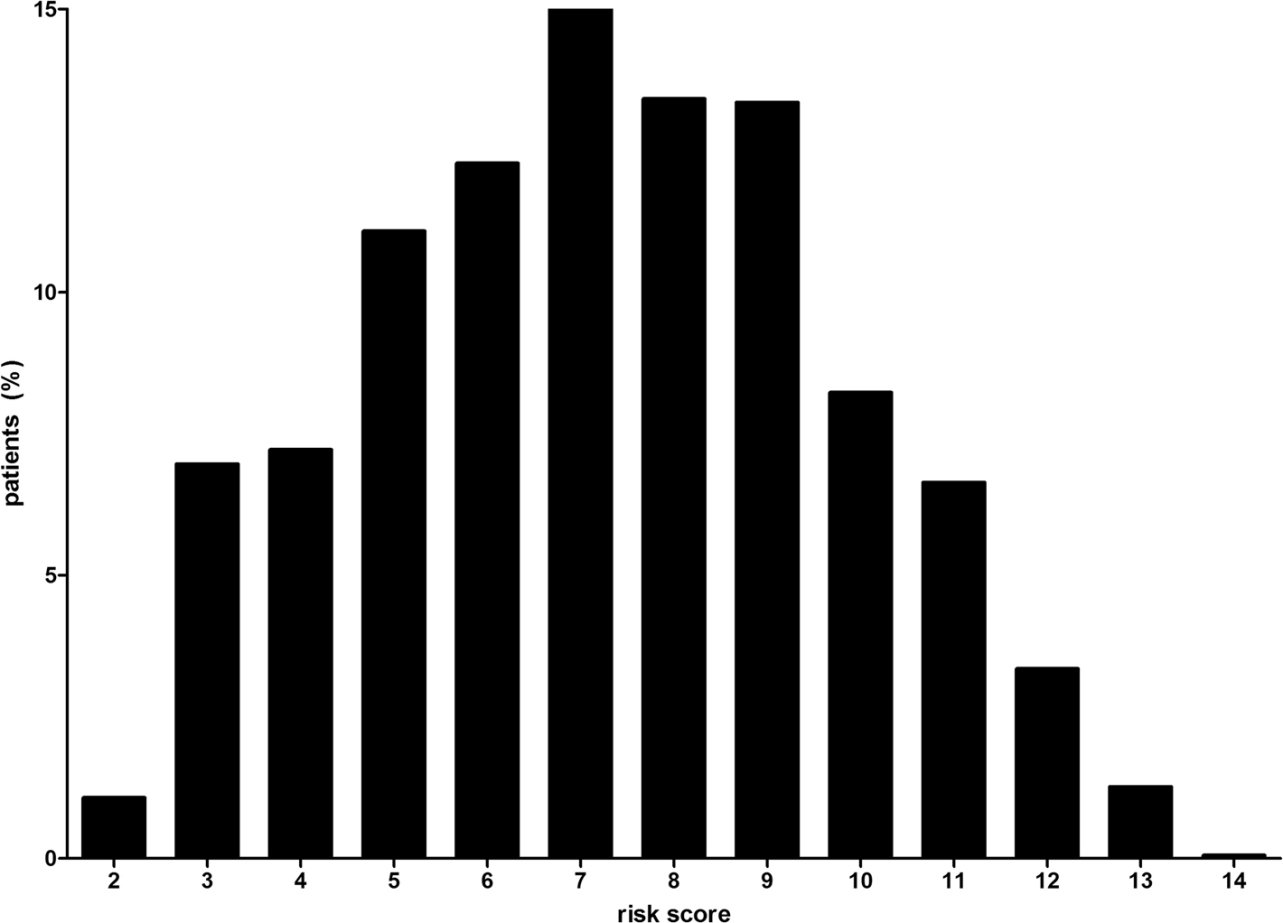
Distribution of the risk scores in the external validation cohort

**Fig. 4:**
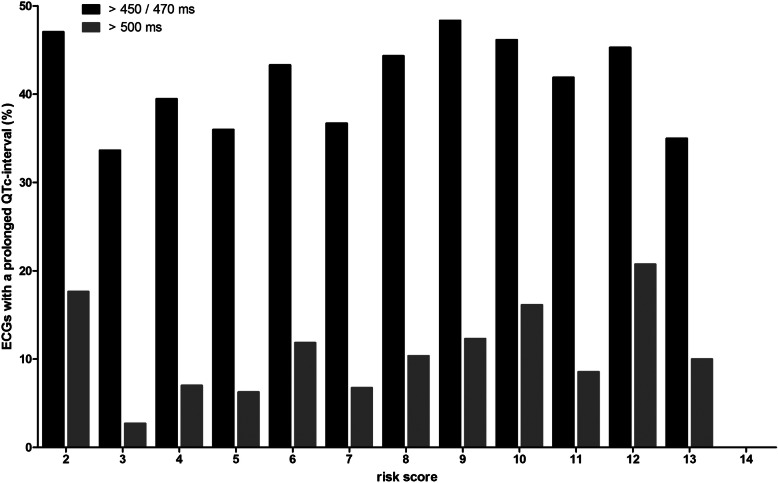
Proportion of ECGs with QTc-prolongation (> 450/470 ms and > 500 ms) versus risk scores in the external validation

The area under the ROC-curve (AUROC) was 0.54 (95% CI 0.51–0.56) as shown in Fig. 2. The performance characteristics of the model are presented in Table 5. The selected optimal cut-off value was 6; 26.3% of all patients scored < 6 points. This cut-off value led to a sensitivity of 76.6% and a specificity of 28.5% to predict patients with a QTc-interval > 450/470 ms (m/f). Figures 3 and 4 show the distribution of the risk scores in the external validation.

**Table 5:** Performance characteristics of the risk model in the external validation when using different cut-off values

Performance characteristics	Cut-off-value ≥ 5		Cut-off value ≥ 6		Cut-off value ≥ 7	
	> 450/470 ms	> 500 ms	> 450/470 ms	> 500 ms	> 450/470 ms	> 500 ms
Sensitivity (%)	86.3	91.0	76.6	83.9	63.8	69.0
Specificity (%)	16.3	15.9	28.5	27.5	40.4	39.5
False Omission Rate (%)	37.3	5.8	36.8	6.0	38.9	7.9
Accuracy (%)	0.45	0.23	0.48	0.33	0.50	0.42
Youden’s index (%)	2.6	6.9	5.1	11.3	4.2	8.5

### Post hoc analysis

In total, 155 ECGs (9.8%) showed a QTc-interval exceeding 500 ms. The mean ± SD risk score of patients with a QTc-interval > 500 ms was 7.9 ± 2.5; the mean ± SD risk score of patients with a normal QTc-interval was 7.2 ± 2.5. The area under the ROC-curve (AUROC) was 0.59 (95% CI 0.54–0.63) as shown in Fig. 2. The cut-off value of 6 led to a sensitivity of 83.9% and a specificity of 27.5% to predict patients with a QTc-interval > 500 ms. Figures 3 and 4 show the distribution of the risk scores in the external validation when QTc-prolongation was defined as > 500 ms.

## Discussion

We have developed a tool which enables the identification of patients with an increased risk of QTc-prolongation when using two or more QTc-prolonging drugs with a known risk of TdP. We chose to develop a tool based on seven predictors, that could easily be implemented in everyday practice. The model was externally validated using an independent dataset of a general teaching hospital, showing the robustness of the model. Implementing such a model in clinical practice might enhance the identification of high-risk patients which will support healthcare providers in selecting patients in whom the risk of QTc-prolongation is such that therapy adjustment and/or additional ECG monitoring is required. At the same time such a model might also identify patients at low risk for developing cardiac arrhythmia, and in whom there is no need for monitoring ECGs after drug initiation, but further improvement of the tool is needed. However, it should be noted that the tool is not designed to fully replace healthcare providers in handling QT drug-drug interactions.

The model, developed in a university medical center, showed insufficient discrimination abilities (AUROC < 0.60) when applied to a dataset from a general teaching hospital. In the development cohort, we used a cut-off value of 450 ms in men and 470 ms in women for a prolonged QTc-interval using the Bazett formula. The Bazett formula often overestimates the QTc-interval in patients with sinus tachycardia [37]. In the development cohort, 91.6% of the patients had heart rates within the range of 60–100 bpm. Arrhythmias are often associated with QTc-intervals exceeding 500 ms [1, 8, 38]. Therefore, we performed a post hoc analysis to compare both reference values. The AUROC curve was 0.54 (95% CI 0.51–0.56) for QTc > 450/470 ms, but increased when QTc-prolongation was defined as QTc > 500 ms (0.59, 95% CI 0.54–0.63). We were aiming for high sensitivities to generate low numbers of false negatives, in order to not miss patients at high risk for TdP. The model was more sensitive in identifying QTc-intervals exceeding 500 ms. The optimized cut-off value of 6 resulted in a sensitivity of 76.6% for the prediction of QTc > 450/470 ms and 83.9% for the prediction of QTc > 500 ms.. However, the low specificity (27.5%) means that the model incorrectly labels patients at risk for QTc-prolongation. Nevertheless, we focused on optimizing sensitivity in order to prevent missing patients at risk for QTc-prolongation by accepting sub-optimal specificity values. As the current guidelines generate alerts in all patients, the guidelines lead to a specificity of zero, so even a specificity of 27.5% is an improvement. A perfect prediction model is not feasible because there is a wide variability in the QTc-interval independent of risk factors. And also, the incidence of QTc-intervals above 500 ms is relatively low [8, 19, 39]. Nevertheless, the sensitivity and specificity value of 83.9 and 27.5% should be optimized before broad implementation in clinical practice can be recommended.

Ideally, the model should be developed and validated with TdP as primary outcome. As linear correlation is lacking, it is questionable whether a prolonged QTc-interval is an adequate marker for predicting the risk of TdP [40]. Unfortunately, it is nearly impossible to identify cases of TdP, because ECGs are frequently not available to ensure TdP actually occurred. Furthermore, even in high risk populations the incidence of TdP is extremely low, so exceptionally large patient populations are needed to study TdP as primary endpoint. So a prolonged QTc-interval is still the most validated and frequently used surrogate marker in clinical practice [2, 41].

Several studies have already introduced risk models for predicting QTc-prolongation/TdP. Haugaa et al. developed the ‘pro-QTc’ risk score, however, the primary endpoint in their study was mortality which is a different endpoint than the primary endpoint used in this study [38]. Tisdale et al. developed a risk score via a similar approach, but included only patients admitted to cardiac care units [15]. Consequently, generalizability to a general population may be limited. Vandael et al. recently developed an optimized RISQ-PATH score to detect high-risk patients for developing QTc-prolongation [42]. However, when this model was applied to patients using two or more QTc-prolonging drugs, the sensitivity of the model was 94.5%, but the specificity of the model was even lower than our model (22.1%). Moreover, the RISQ-PATH score of Vandael et al. consists of too many predictors which are frequently not available and, therefore, this tool cannot be used in clinical practice. In addition, this tool needs to be implemented in the clinical decision support system before it is applicable in primary care, which does not seem feasible with the current electronic patient health records. We aimed to develop a risk score to detect high-risk patients when using two or more QTc-prolonging drugs which is easily applicable in both primary and hospital care.

A major strength of this study is that we externally validated the risk model in an independent dataset from a general teaching hospital. External validations are able to determine the generalizability of predicting models in different settings [43].

Several limitations of our study need to be addressed. First, the study was limited by a single-center design for model development; however, patients were admitted to all general nursing departments representing a general hospital population. Second, the sample size was relatively small which increased the risk of model overfitting; a common problem in models derived from small datasets. By validating the model in a large external dataset and by adding predictors based on a review of literature, the risk of overfitting was minimized [25, 26]. In the validation cohort, there might have been selection bias as the prevalence of QTc-prolongation (41.5%) was quite high compared to the overall prevalence found in the literature review (21%). We retrospectively collected these data, so presumably, ECGs were mainly recorded in high-risk patients. Our model does not take into account the QTc-interval at baseline. Given that the risk on QTc-prolongation increases when a high baseline QTc-interval is present, we chose to exclude this potential predictor because baseline ECGs are frequently not available in clinical practice. Also, the small dataset precluded the inclusion of too many predictors in the model. Third, the tool does not take into account the variety of QT-DDIs as our aim was to develop an easily obtainable model that can be used in different healthcare settings. Because of the different pharmacological pathways of the QTc-prolonging drugs via inhibition of the hERG channels or Cytochrome P450 enzymes, stratification of QT-DDIs is extremely complex and larger studies need to be conducted for each QT-DDI separately [44, 45].

The performance characteristics of the model were not perfect. Also after performing a post-hoc analysis, the discrimination ability of the model remained limited. This can be explained by the discrepancies between the development and validation cohort. First, the validation dataset included patients from all departments including ICU patients, whereas the development dataset only included patients from medical wards. Unfortunately, we could not exclude these patients in the validation dataset, because it was unknown to which department patients had been admitted. Therefore, we decided to exclude patients using propofol in order to exclude perioperative and ICU patients as much as possible. Also, patients with ICDs or ECG abnormalities were not excluded in the validation cohort because these data could not be extracted. Therefore, we excluded ECGs with deviant heart rates and QTc-intervals. We did correct the QT-interval for wide QRS-complexes to limit ECG exclusions. Second, the QTc-intervals of the development cohort were manually measured, while the QTc-intervals of the validation cohort were automatically calculated by the MUSE Cardiology Information System. But most importantly, the retrospective design of the external validation where only patients in whom an ECG was recorded during use of the QTc-prolonging drugs were included, may have led to selection bias. ECGs are more likely to be recorded in vulnerable patients. According to the high prevalence of comorbidities in the validation cohort, this was probably the case. But even in high risk populations, QTc-prolongation is not always present resulting in false positives. Also, ECGs are more likely to be recorded in patients with underlying cardiac diseases or with suspected QTc-prolongation even if they only have a few risk factors, resulting in false negatives. Our preliminary results must therefore be confirmed in large studies where this selections bias is not present. The usability of the tool must be evaluated in a clinical setting. For future perspectives, this tool must be further studied to assess its effect when it is integrated in an electronic decision support system before implementation can be recommended. A clinical decision support system is extensively used by pharmacists, as it is part of their job to read DDI alerts. Ideally, the system will automatically calculate a risk score for the individual patient and only generate alerts in high-risk patients resulting in more specific alerts. Such a study should be performed in large patient groups with clinically relevant endpoints.

## Conclusion

To conclude, we developed and validated a tool to predict patients at risk for QTc-prolongation when using two or more QTc-prolonging drugs. The model is able to predict patients at risk for QTc-prolongation (> 500 ms) with a sensitivity of 83.9% and specificity of 27.5% at an optimized cut-off value of 6. This tool might contribute to support the risk management of QT-DDIs in clinical practice, but further testing of the tool is needed in study cohorts without any selection bias. Eventually, a clinical decision support tool will support healthcare providers in selecting patients in whom monitoring ECGs or switching therapy can be withheld, without compromising patient safety.

## Data Availability

The datasets used and/or analysed during the current study are available from the corresponding author on reasonable request.

## Abbreviations

AUROC: Area under the ROC-curve
AZCERT: Arizona center for education and research on therapeutics
BPM: Beats per minute
95% CI: 95% Confidence intervals
CPOE: Computerized physician order entry
C-statistic: Concordance statistic
ECG: Electrocardiogram
eGFR: Estimated glomerular filtration rate
ICD: Implantable cardioverter-defibrillator
ICU: Intensive care unit
IQR: Interquartile range
LBBB/RBBB: Left or right bundle branch block
MDRD: Modification of diet in renal disease
OR: Odds ratios
QT-DDI: QT drug-drug interaction
ROC: Receiver operating characteristics
SPSS: Statistical package for social science
SD: Standard deviation
TdP: Torsade de pointes
VR: Ventricular rate
